# Supplementary material for: Altered microRNA Transcriptome in Cultured Human Liver Cells upon Infection with Ebola Virus
Source: Int J Mol Sci. 2021 Apr 6;22(7):3792. doi: 10.3390/ijms22073792 (PMC8038836; doi:10.3390/ijms22073792)
Supplement: Supplementary file 1 [file ijms-22-03792-s001.zip › Supplementary_File/C_ GO_Analysis_Results/16-30nt_go_Reston-24h-Huh7_vs_Control-24h-Huh7_up.mature_mirna_targets/MF_Pvalue_tree.pdf]

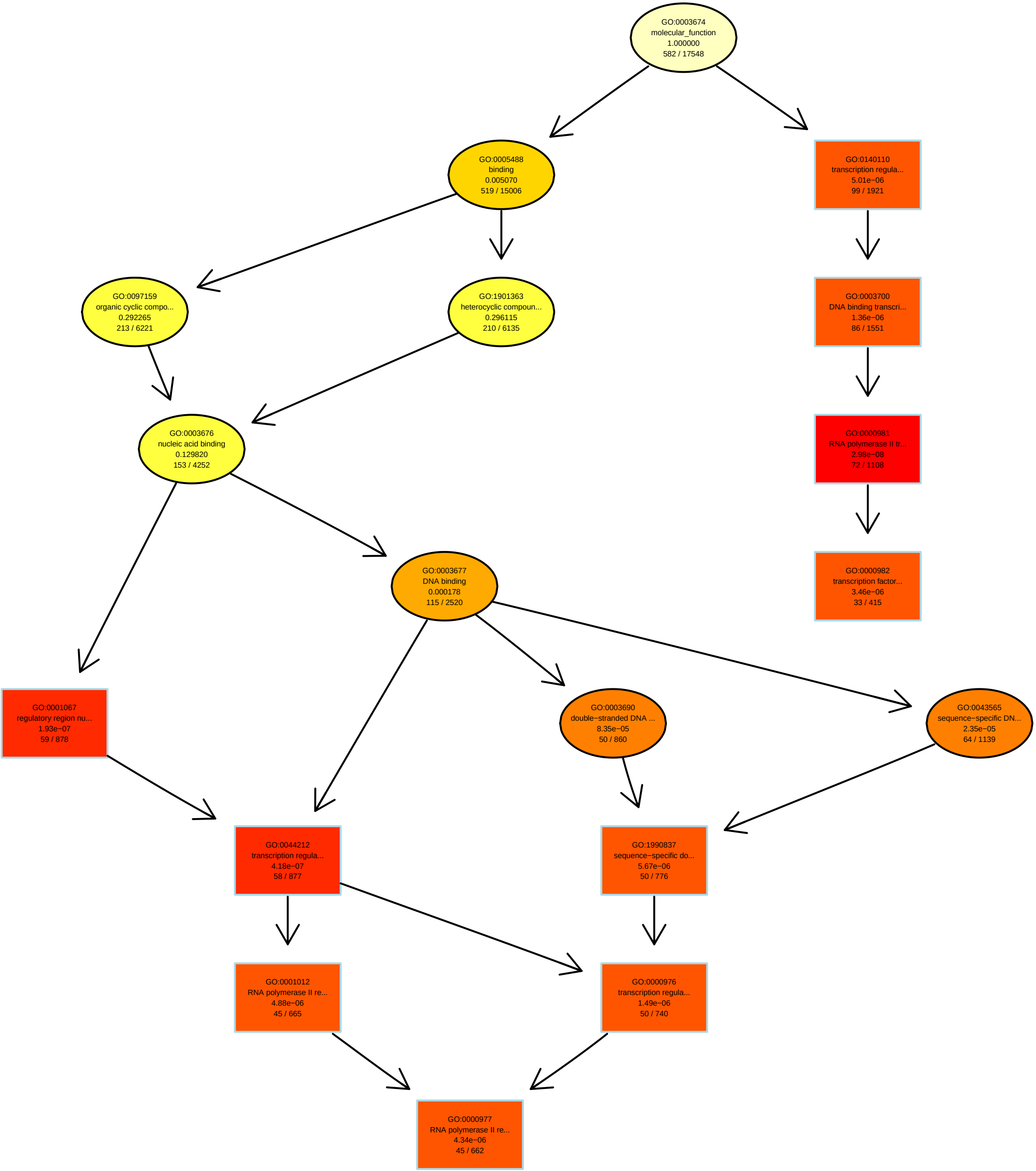
GO:0003674
molecular_function
1.000000
582 / 17548
GO:0005488
binding
0.005070
519 / 15006
GO:0140110
transcription regula...
5.01e−06
99 / 1921
GO:0097159
organic cyclic compo...
0.292265
213 / 6221
GO:1901363
heterocyclic compoun...
0.296115
210 / 6135
GO:0003700
DNA binding transcri...
1.36e−06
86 / 1551
GO:0003676
nucleic acid binding
0.129820
153 / 4252
GO:0000981
RNA polymerase II tr...
2.98e−08
72 / 1108
GO:0003677
DNA binding
0.000178
115 / 2520
GO:0000982
transcription factor...
3.46e−06
33 / 415
GO:0001067
regulatory region nu...
1.93e−07
59 / 878
GO:0003690
double−stranded DNA ...
8.35e−05
50 / 860
GO:0043565
sequence−specific DN...
2.35e−05
64 / 1139
GO:0044212
transcription regula...
4.18e−07
58 / 877
GO:1990837
sequence−specific do...
5.67e−06
50 / 776
GO:0001012
RNA polymerase II re...
4.88e−06
45 / 665
GO:0000976
transcription regula...
1.49e−06
50 / 740
GO:0000977
RNA polymerase II re...
4.34e−06
45 / 662
